# Exercise Ameliorates Dopaminergic Neurodegeneration in Parkinson’s Disease Mice by Suppressing Microglia-Regulated Neuroinflammation Through Irisin/AMPK/Sirt1 Pathway

**DOI:** 10.3390/biology14080955

**Published:** 2025-07-29

**Authors:** Bin Wang, Nan Li, Yuanxin Wang, Xin Tian, Junjie Lin, Xin Zhang, Haocheng Xu, Yu Sun, Renqing Zhao

**Affiliations:** College of Physical Education, Yangzhou University, Yangzhou 225009, China; bwang1999@126.com (B.W.); 15151119732@163.com (N.L.); wangyuanxin880330@163.com (Y.W.); tianxin96331@163.com (X.T.); junjielin2022@163.com (J.L.); xxinz1998@163.com (X.Z.); haochengxu2023@163.com (H.X.); 15050599442@163.com (Y.S.)

**Keywords:** exercise, irisin, microglia, neuroinflammation, apoptosis, Parkinson’s Disease

## Abstract

Although exercise is known to exert anti-inflammatory effects in neurodegenerative diseases, its specific impact and underlying mechanisms in Parkinson’s disease (PD) remain poorly understood. This study explores the effects of exercise on microglia-mediated neuroinflammation and apoptosis in a PD model, focusing on the role of irisin signaling in mediating these effects. Using a 1-methyl-4-phenyl-1,2,3,6-tetrahydropyridine (MPTP)-induced PD mouse model, we found that a 10-week treadmill exercise regimen significantly enhanced motor function, reduced dopaminergic neuron loss, attenuated neuronal apoptosis, and alleviated neuroinflammation. Exercise also shifted microglia from a pro-inflammatory to an anti-inflammatory phenotype. Notably, levels of irisin, phosphorylated AMP-activated protein kinase (p-AMPK), and sirtuin 1 (Sirt1), which were decreased in the PD brain, were significantly increased following exercise. These beneficial effects were abolished by blocking the irisin receptor with cyclic arginine–glycine–aspartic acid–tyrosine–lysine (cycloRGDyk). Our results indicate that exercise promotes neuroprotection in PD by modulating microglial activation and the AMPK/Sirt1 pathway through irisin signaling, offering new insights into exercise-based therapeutic approaches for PD.

## Simple Summary

This study demonstrates that 10 weeks of exercise improves motor function and protects dopamine-producing neurons in a Parkinson’s disease (PD) mouse model. Exercise reduces brain inflammation by shifting microglia (immune cells in the brain) from an inflammatory state to a protective state. These benefits are linked to increased levels of irisin, a muscle-derived hormone, which activates the AMPK/Sirt1 pathway—a key regulator of energy metabolism and inflammation. Blocking irisin’s receptor reversed these positive effects, confirming its role in exercise-induced neuroprotection. These findings suggest that exercise, through irisin signaling, may help slow PD progression by reducing inflammation and neuronal damage.

## 1. Introduction

PD is a degenerative condition that affects older adults globally [1]. In addition to its common motor symptoms like tremors, muscle stiffness, slow movement, and problems with posture and walking, the disease can also lead to cognitive issues, sleep disturbances, and sensory problems as it advances [2]. After being diagnosed with PD, patients usually have a life expectancy of around 7 to 15 years on average [3]. According to reports, the disease caused the death of about 117,400 people globally in 2015 [4]. More importantly, it is predicted that the number of people over 50 who suffer from PD will double by 2030 [5]. PD is characterized by the gradual loss of dopamine (DA) in the brain region that controls movement (substantia nigra (SN)–striatum (ST) pathway) and the accumulation of α-synuclein, which results in the formation of Lewy bodies (LBs) in the cytoplasm of the remaining neurons in the SN [2]. As the disease progresses, conventional medications become less effective and may cause some side effects such as edema, joint stiffness, and hallucinations [3,6]. Consequently, there is an urgent need for a better comprehension of the disease’s pathology and the development of effective intervention strategies.

Growing evidence has indicated that neuroinflammation, which involves the activation of glial cells and the infiltration of various peripheral immune cells, plays a significant role in the complex pathogenesis of PD [7]. This leads to a subsequent release of pro-inflammatory cytokines, chemokines, and other harmful chemicals, resulting in damage to the blood–brain barrier (BBB) and excessive neuronal cell apoptosis [8]. As a result, neuronal degeneration and loss are increased, which further worsens the cerebral inflammatory condition and tissue injury [9]. Microglia are highly dynamic immune cells that exhibit remarkable plasticity, dynamically adapting their morphological, transcriptional, and functional states in response to spatiotemporal cues in the central nervous system (CNS) [10]. While traditionally described in the context of a simplified M1/M2 dichotomy, emerging evidence emphasizes that microglial activation exists along a continuum of states rather than as discrete phenotypes [11]. For instance, microglia can sense brain disturbances—such as injury, infection, or protein aggregation—and adapt their responses along a continuum of states, ranging from pro-inflammatory activity to tissue repair mechanisms. This plasticity is critical: during pathogen clearance, microglia may upregulate pro-inflammatory markers like inducible nitric oxide synthase (iNOS), cluster of differentiation 16 (CD16), and cluster of differentiation 11b (CD11b), while in regenerative contexts, they promote tissue repair via markers like cluster of differentiation 206 (CD206), chitinase-like protein 3 (YM-1), and arginase-1 (Arg-1) [12,13,14]. However, the binary M1/M2 framework fails to capture the full spectrum of microglial responses, which are better characterized by dynamic transcriptional states and functional modules. Postmortem studies of PD patients reveal microglial reactivity in the substantia nigra and striatum, characterized by increased expression of inflammatory markers and reduced neuroprotective signals, which may contribute to dopaminergic neuron degeneration [15,16]. These findings suggest that modulating microglial reactivity could offer therapeutic potential for alleviating PD neuropathology.

Recent evidence suggests that regular exercise can provide numerous benefits to the brain [17,18], such as improving neuroinflammation, reducing neuronal apoptosis, and alleviating dopaminergic neuron loss in animal models of PD [19,20]. For example, a four-week treadmill exercise program was shown to markedly improve motor dysfunction and reduce the loss of DA neurons in the SN and ST of ICR PD mice [21]. Additionally, exercise intervention in PD mice significantly reduced the plasma membrane expression of CD11b, while upregulating the anti-inflammatory marker cluster of differentiation 200 (CD200) and its receptor compared to sedentary controls [21]. However, the precise molecular mechanisms through which exercise exerts its beneficial effect on PD pathology remain unclear.

The capacity of exercise to improve an individual’s health is regarded partly by its production of various myokines, such as irisin, which is a cleavage product of fibronectin type III domain-containing protein 5 (FNDC5) [22,23]. These myokines can circulate in the blood and enter various tissues, including the brain, where they exert their functions [22,24,25]. Recently, the existence of irisin was determined in diverse regions and cell populations of the brain [22]. The evidence that alterations in irisin expression play a role in reducing neuroinflammation and excessive apoptosis highlights its importance in maintaining brain plasticity and function [23,26,27]. More importantly, irisin plays a critical role as a linking molecule in the interaction between exercise and neuropathology and cognition in PD as well as other neurodegenerative diseases [23,28,29,30]. Irisin frequently interacts with other myokines or factors to facilitate the advantageous effects of exercise on neuroinflammation and cognitive performance. AMPK is a crucial molecule involved in exercise intervention that has a significant impact on neuronal functions. The phosphorylation of AMPK promotes microglial polarization toward anti-inflammatory programs, reduces neuroinflammation, and alleviates excessive apoptosis in mice with brain injuries [31]. Sirt1, a downstream molecule of AMPK, is responsible for regulating several important signaling pathways, including apoptosis, oxidative stress, and inflammation [32]. Studies have shown that Sirt1 can alleviate chronic and acute neurological disorders by modulating particular transcription factors through deacetylation [33]. Recent evidence has shown that during exercise, irisin, AMPK, and Sirt1 cooperate to control cell metabolism and immune activity [34]. However, their combined role in improving neuroinflammation and cognitive function in response to exercise needs to be addressed.

The potential role of irisin in combination with AMPK and Sirt1 in regulating neuroinflammation during exercise suggests that it could be a promising target for combating PD through exercise intervention. However, the exact mechanism by which irisin exerts its neuroprotective effects against neuroinflammation during exercise remains unclear, and further validation is needed to determine whether irisin plays a central role in the beneficial effects of exercise on PD. To address this, we induced the PD mice model using MPTP, administered via intraperitoneal injection for 5 consecutive days—a protocol that recapitulates key PD features including dopaminergic neurodegeneration and motor dysfunction [35]. This study aimed to investigate whether exercise-induced irisin could modulate the AMPK/Sirt1 signaling pathway, ultimately leading to the attenuation of microglia-mediated neuroinflammation and excessive apoptosis in MPTP-induced mice, and potentially ameliorating PD.

## 2. Materials and Methods

### 2.1. Animals and Protocols

All methods are reported under ARRIVE guidelines [36]. Thirty-two male C57BL/6 mice, aged 2 months and weighing 26 ± 2 g, were obtained from the Experimental Animal Center of Yangzhou University (Jiangsu, China). The mice were housed in cages in controlled environmental conditions, including a temperature of 24 ± 2 °C, humidity of 55 ± 6%, and a 12:12 h light–dark cycle. This study was conducted following the guidelines set by the Yangzhou University Animal Ethics Committee (No. 202303133), ensuring strict adherence to those concerning the care and use of laboratory animals.

After one week of acclimation, the mice were assigned at random to either the control group (CON, *n* = 8) or one of three experimental groups that received MPTP injections to create PD models. The experimental mice were either sedentary (PD, *n* = 8), performed 10 weeks of exercise (PE, *n* = 8), or received 10 weeks of exercise and cycloRGDyk (inhibitors of irisin receptor) interventions (PERG, *n* = 8), a group used to verify whether irisin is a key mediator of the neuroprotective effects exerted by exercise.

After the completion of the behavioral test following the final exercise and drug intervention, mice were anesthetized with a dose of urethane (1.5 g/kg, intraperitoneally). Once a deep level of anesthesia was confirmed, euthanasia was performed to collect brain samples. The left brain hemisphere of the mice was fixed in 4% paraformaldehyde for staining, while the right hemisphere was dissected to isolate the SN region according to the stereotaxic atlas. The dissected substantia nigra tissue was rapidly frozen in liquid nitrogen to preserve dopaminergic neuron-rich regions for molecular analyses.

### 2.2. PD Model Development and Drug Treatment

MPTP, a neurotoxin inhibiting mitochondrial complex I to induce dopaminergic neuron degeneration, is widely used to establish PD animal models [35]. Notably, the MPTP-induced PD mouse model effectively recapitulates human PD’s behavioral, neurochemical, and neuropathological features, making it suitable for PD and neuroinflammation research. In this study, we dissolved MPTP (Sigma-Aldrich, St. Louis, MO, USA) in 0.9% saline at a concentration of 3.0 mg/mL and injected it intraperitoneally 30 mg·kg^−1^·d^−1^ for 5 days.

For irisin antagonists, we chose the irisin receptor inhibitor cycloRGDyk (GLPBIO Biological Company, Montclair, CA, USA), as studied by Kim et al. [37]. The cycloRGDyk dissolved in DMSO was administered intravenously via the tail at 2.5 mg/kg twice weekly for 10 weeks [38].

### 2.3. Treadmill Running Protocol

Over 10 weeks, a 0° inclination aerobic treadmill was used 5 times per week. During the first week of the acclimatization exercise, the running pace was gradually increased from 8 to 12 m/min, and the exercise duration was gradually extended from 10 to 60 min. For the following 9 weeks, the mice exercised for 60 min every workout day, maintaining a pace of 12 m/min [19].

### 2.4. Rotarod Test

The balanced locomotor performance of the mice was evaluated using a rotating apparatus (Ugo Basile, Gemonio, Italy). All mice were pre-trained by being placed on a spinning rod for 3 consecutive days. In the formal test, after the mice were positioned, the initial speed of the rotating bar was set to 1 rpm, and the test was run using an acceleration mode of 12 rpm/2 s until the speed was increased to 50 rpm [39]. The time the mouse spent on the rod was recorded for a maximum of 300 s. The results were determined as the average of three replicate experiments, each of which was conducted 40 min apart.

### 2.5. Enzyme-Linked Immunosorbent Assay (ELISA)

Mouse serum irisin levels were measured using a monoclonal antibody-based ELISA kit (JL46388-96T, detection range: 15.6–1000 pg/mL; sensitivity: 7.7 pg/mL), following the standard operating procedures provided with the kit (Jianglaibio, Shanghai, China).

### 2.6. Western Blot Analysis

Mice SNs were homogenized in RIPA lysis buffer (P0013B, Beyotime) containing 1% protease inhibitor cocktail (P8340, Sigma-Aldrich) to obtain total protein. Western blotting was performed using SDS-PAGE. Briefly, PVDF membranes (LAV0045, Horizone Technologies, WideView Biotech Co., Ltd., Hangzhou, China) were blocked with 10% skimmed milk (Cat# 1706404, Bio-Rad, Hercules, CA, USA) (10 g non-fat dry milk powder prepared in TBST) for 2 h at room temperature with gentle agitation. The irisin (1:1500, SRP8039, Merck, Darmstadt, Germany), AMPK (1:1000, AF6195, Beyotime, Shanghai, China), p-AMPK (1: 2000, AF5908, Beyotime, China), Sirt1 (1:500, sc-74465, Santa, Dallas, TX, USA), IL-1β (1:2000, AF5103, Affinity, Cincinnati, OH, USA), ionized calcium-binding adapter molecule 1 (Iba-1, 1:1000, AF7143, Beyotime), TNF-α (1:500, sc-12744, Santa), B-cell lymphoma-2-associated X protein (Bax, 1:1000, WL01637, Wanlei, Shanghai, China), B-cell lymphoma-2 (Bcl-2, 1:1000, WL01556, Wanlei), and β-actin (1:50,000, 66009-1-lg, Proteintech, Rosemont, IL, USA) antibodies were applied in 10% skimmed milk overnight at 4 °C, followed by goat anti-rabbit IgG (1:200, AS014, Abclonal, Woburn, MA, USA) or goat anti-mouse IgG (1:200, G1214-100UL, Servicebio, Wuhan, China) for 1 h. The samples were derived from the same experiment, and blots were processed in parallel. The density of protein bands was evaluated with Image J software (version 6.0, Media Cybernetics, Rockville, MD, USA) and normalized to the equivalent density of the housekeeping protein β-actin [40].

### 2.7. Immunohistochemistry and Immunofluorescence Staining

Coronal sections (4 μm) of paraffin-embedded left hemispheres were cut using a microtome (Leica RM2235, Leica Biosystems, Nussloch, Germany), targeting the SN (−2.92 to −3.80 mm from bregma) and ST (+0.98 to −0.10 mm) with reference to a mouse brain atlas. Peroxidase blocking employed 3% H_2_O_2_ for 10 min. The tyrosine hydroxylase (TH) antibody (1:500, GB11181, Servicebio) was applied in 5% skimmed milk (Cat# 1706404, Bio-Rad) (5 g non-fat dry milk powder prepared in TBST) overnight at 4 °C, followed by HRP-conjugated goat anti-rabbit secondary antibody (1:200, GB23303, Servicebio, Wuhan, China) (diluted in 5% skimmed milk) for 40 min at room temperature. The procedure for immunofluorescence staining is the same as described above, except that the cysteine–aspartic protease-3 (caspase-3, 1:50, sc-56053, Santa), TH (1:500, GB11181, Servicebio), Iba-1 (1:100, AF7143, Beyotime), IL-1β (1:200, AF5103, Affinity), TNF-α (1:50, sc-12744, Santa), iNOS (1:50, sc-7271, Santa), and CD206 (1:50, sc-58986, Santa) primary antibodies and Alexa Fluor 488-conjugated goat anti-rabbit IgG (1:200, GB25303, Servicebio) and Cy5-conjugated goat anti-mouse IgG (1:200, GB27301, Servicebio) fluorescence-labeled secondary antibodies were used. Nuclear counterstaining was performed by incubating sections with DAPI (G1012, Servicebio) for 10 min in the dark. Images were acquired using a Nikon Eclipse Ni-U microscope with a DS-Ri2 camera (Nikon, Tokyo, Japan). For optimal visualization, brightness was uniformly adjusted across all image panels using ImageJ software (version 1.54 g, National Institutes of Health, Bethesda, MD, USA). Subsequent quantitative analysis was performed using the same software.

### 2.8. Real-Time Quantitative Polymerase Chain Reaction (RT q-PCR)

Total RNA was extracted from microdissected SN tissue using TRIzol reagent (Invitrogen, Carlsbad, CA, USA), with concentration and purity (A260/A280: 1.8–2.0) measured by NanoDrop 2000. cDNA was synthesized from 1 μg RNA using PrimeScript RT Master Mix (Takara, Kusatsu, Shiga, Japan). qPCR was performed in 20 μL reactions containing ChamQ SYBR Master Mix (Vazyme, Nanjing, China), 200 nM primers (sequences in Table 1; designed via Allele ID 7.5 and validated by BLAST/melt curve analysis), and 50 ng cDNA template, using a QuantStudio 5 system (Applied Biosystems, Foster City, CA, USA) under standard cycling conditions (95 °C/30 s, 40 cycles of 95 °C/10 s, and 60 °C/30 s). Data were normalized to GAPDH (ΔΔCt method), with primer efficiencies (90–110%) confirmed by standard curves.

### 2.9. Statistical Analysis

GraphPad Prism 9.4.1 (GraphPad Software, Boston, MA, USA) was used to analyze the experimental data, and the results are presented as mean ± standard deviation (SD). Normality and homogeneity of variances were assessed using the Shapiro–Wilk test and Levene’s test for equality of variances, respectively. For normally distributed data with homogeneous variances, two-group comparisons were analyzed using the independent samples *t*-test, while multiple-group comparisons were performed using one-way ANOVA followed by Tukey’s post hoc test. For non-normally distributed data, the Mann–Whitney U test was used for two-group comparisons, and the Kruskal–Wallis test with Dunn’s post hoc test was applied for multiple-group comparisons. When variances were heterogeneous, Welch’s corrected *t*-test or Brown–Forsythe ANOVA was used for parametric analyses. All statistical tests were two-tailed, and significance was set at *p* < 0.05.

## 3. Results

### 3.1. MPTP-Induced Locomotor Impairment and the Loss of Dopaminergic Neurons Are Improved by Exercise Intervention

**Figure 1:**
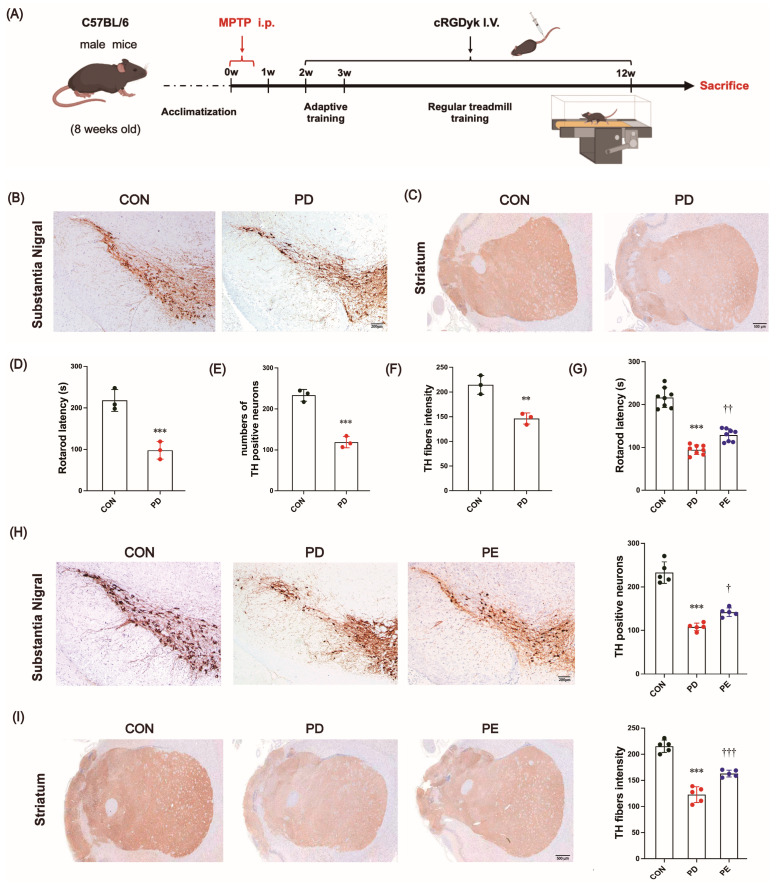
(**A**) Schematic overview of animal experimental procedures. (**B**,**C**) Representative images of TH expression in the substantia nigra (Scale bars, 200 μm) and striatum (Scale bars, 500 μm). (**D**) The amount of time the mice stayed on the pole in the rotarod test after MPTP injection. (**E**,**F**) Quantitative analysis of TH staining in the substantia nigra and striatum. (**G**) The amount of time the mice stayed on the pole in the rotarod test after all interventions. (**H**,**I**) Representative images of TH expression in the substantia nigra (Scale bars, 200 μm) and striatum (Scale bars, 500 μm), and relative quantitative analysis. ** and *** represent *p* < 0.01 and *p* < 0.001, respectively, when comparing with the CON group; ^†^, ^††^, and ^†††^ indicate *p* < 0.05, *p* < 0.01, and *p* < 0.001, respectively, in comparison with the PD group. CON, control; PD, Parkinson’s disease; PE, PD plus exercise.

The PD mouse model was generated by intraperitoneal injection of MPTP as previously described [41] (Figure 1A). After inducing PD-like symptoms, MPTP-treated mice exhibited a much shorter time on the rod than the untreated mice in the rotarod test (Figure 1D, *p* < 0.001). To assess the loss of dopaminergic neurons, we conducted immunostaining of these neurons using the TH antibody, which could effectively label and measure the number of TH-immunoreactive neurons [42]. The PD mice exhibited a notable decrease in TH immunoreactivity in the SN (*p* < 0.001) and ST (*p* = 0.006) regions compared to the CON group (Figure 1B,C,E,F, negative control images of TH immunohistochemical staining in the substantia nigra and striatum are shown in Figure S1). These findings indicate that the MPTP treatment was effective in generating the PD model.

Previous studies have reported conflicting results regarding the impact of exercise on motor function and TH staining [43]. We, therefore, next investigated whether regular exercise could improve motor dysfunction and prevent neuropathological progression. After 10 weeks of treadmill exercise, the PD mice exhibited a significant improvement in their retention time during the rotation test (Figure 1G, *p* = 0.002), as well as a notable increase in TH immunoreactivity observed within the SN (*p* = 0.015) and ST (*p* < 0.001) regions compared to the sedentary PD mice (Figure 1H,I). Overall, exercise has the potential to improve motor function impairment and alleviate dopaminergic neurodegeneration in PD.

### 3.2. Exercise Reduces Excessive Apoptosis in Nigrostriatal Neurons of PD Mice

In healthy individuals, dopaminergic neurons account for approximately 70–80% of all cell types in the SN [44]. Previous studies have detected extensive apoptosis in the midbrain of PD patients [45,46] and suggested that excessive apoptosis may be the primary cause of dopaminergic neuron loss in the SN [47,48]. Therefore, our next objective was to examine cellular apoptosis in PD mice and verify whether the principal apoptotic cells were dopaminergic neurons.

**Figure 2:**
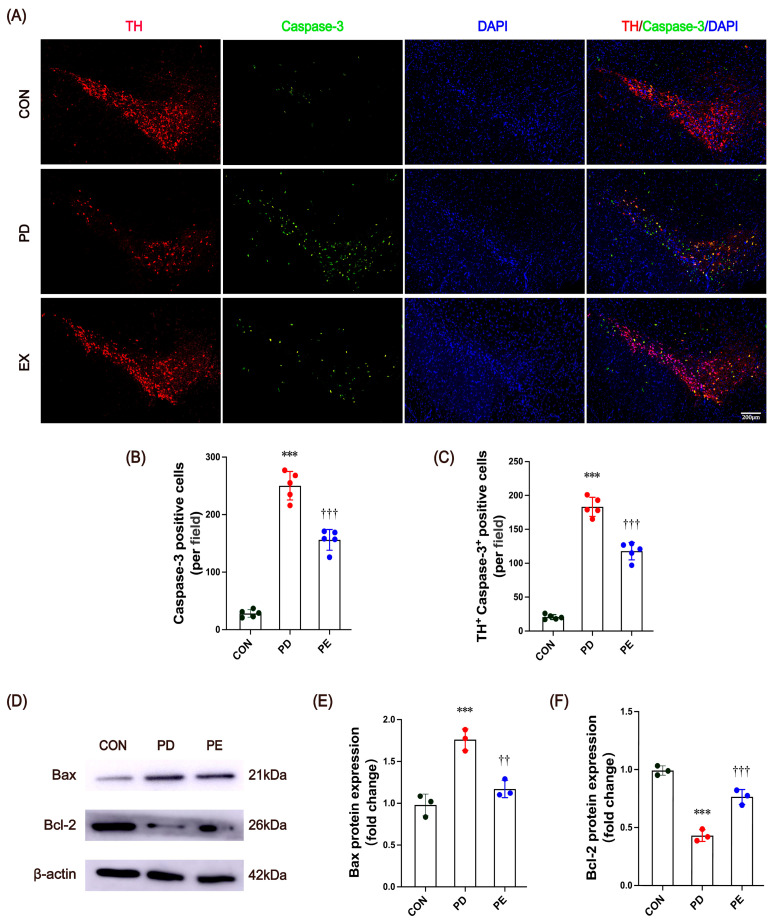
(**A**) Representative images and enlargements of fluorescent double staining of TH (red), caspase-3 (green), and DAPI (blue) (scale bars, 200 μm). (**B**) Quantitative analysis of caspase-3 staining. (**C**) Number of co-localized TH^+^ caspase-3^+^ cells (per field). (**D**) Western blot bands of Bax and Bcl-2 protein in mice substantia nigra. (**E**,**F**) Quantitative analysis of Bax and Bcl-2 proteins. All immunofluorescence images were processed with uniform brightness/contrast adjustments using ImageJ software without altering the original data interpretation. *** represent *p* < 0.001 when comparing with the CON group; ^††^ and ^†††^ indicate *p* < 0.01 and *p* < 0.001, respectively, in comparison with the PD group. CON, control; PD, Parkinson’s disease; PE, PD plus exercise.

Compared with the control mice, the PD mice exhibited a significant increase in caspase-3-positive cells (Figure 2A,B; *p* < 0.001), along with markedly elevated numbers of caspase-3/TH co-localized cells (Figure 2A,C, *p* < 0.001). Ten weeks of exercise training significantly reduced both caspase-3-positive cells (Figure 2A,B; *p* < 0.001) and caspase-3/TH co-localized cell counts (Figure 2A,C, *p* < 0.001). Furthermore, excessive apoptosis is typically accompanied by altered expression of apoptosis-related proteins, such as increased levels of the pro-apoptotic protein Bax and decreased expression of the anti-apoptotic protein Bcl-2 [49]. Our results demonstrated significantly enhanced Bax immunoreactivity and reduced Bcl-2 immunoreactivity in PD mice (Figure 2D–F; *p* < 0.001). Notably, 10-week treadmill exercise intervention reversed Bax (Figure 2D,E; *p* = 0.007) and Bcl-2 (Figure 2D,F; *p* < 0.001) protein expression. These findings indicate that MPTP-induced PD mice develop substantial dopaminergic neuron apoptosis, and importantly, exercise can prevent excessive apoptosis and modulate the associated protein expression profiles in PD.

### 3.3. Exercise Inhibits Microglia Activation and Inflammatory Factor Expression in the Substantia Nigra of PD Mice

**Figure 3:**
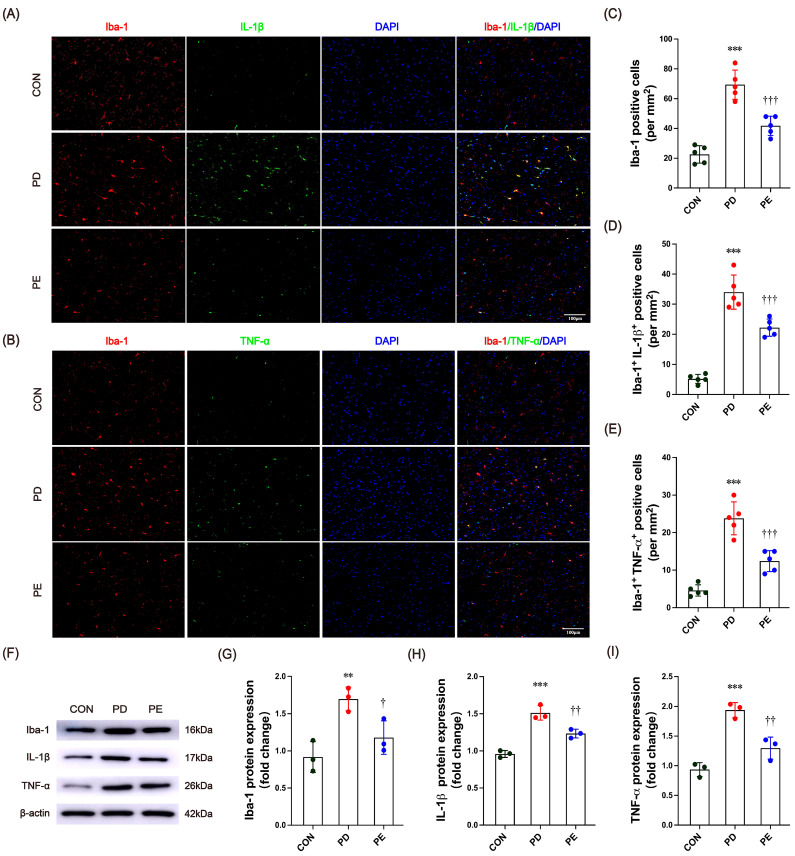
(**A**,**B**) Representative images of Iba-1 (red), pro-inflammatory cytokine IL-1β and TNF-α (green), and DAPI (blue) immunofluorescence staining (scale bars, 100 μm). (**C**) Quantitative analysis of Iba-1 staining. (**D**) Number of co-localized Iba-1^+^ IL-1β ^+^ cells (mm^2^). (**E**) Number of co-localized Iba-1^+^ TNF-α^+^ cells (mm^2^). (**F**–**I**) Western blot bands of Iba-1, IL-1β, and TNF-α protein in mice substantia nigra and quantitative analysis. All immunofluorescence images were processed with uniform brightness/contrast adjustments using ImageJ software without altering the original data interpretation. ** and *** represent *p* < 0.01 and *p* < 0.001, respectively, when comparing with the CON group; ^†^, ^††^, and ^†††^ indicate *p* < 0.05, *p* < 0.01, and *p* < 0.001, respectively, in comparison with the PD group. CON, control; PD, Parkinson’s disease; PE, PD plus exercise.

Recent findings suggest that neuroinflammation is a hallmark of PD pathology. Increased production of inflammatory cytokines may contribute to excessive cell apoptosis and the loss of dopamine-producing neurons [50]. Therefore, we proceeded to investigate the changes in immune activity and markers related to inflammation in the SN of mice with PD. Immunofluorescence staining analysis revealed that compared to the control group, PD-model mice exhibited significantly increased expression of the microglial marker Iba-1 (Figure 3A–C, *p* < 0.001), along with enhanced co-localization of Iba-1 with the pro-inflammatory cytokines IL-1β (Figure 3A,D, *p* < 0.001) and TNF-α (Figure 3B,E, *p* < 0.001). Following the 10-week treadmill exercise intervention, the number of Iba-1-positive cells (Figure 3A–C, *p* < 0.001) as well as Iba-1^+^ IL-1β^+^ (Figure 3A,D, *p* < 0.001) and Iba-1^+^TNF-α^+^ (Figure 3B,E, *p* < 0.001) co-labeled neurons were significantly reduced in PD mice. These findings suggest that regular exercise training may alleviate neuroinflammation associated with PD by suppressing microglial activation and downregulating pro-inflammatory cytokine expression. In addition, Western blot analysis revealed a significant increase in the protein expression of Iba-1 (*p* = 0.007) and inflammatory factors IL-1β (*p* < 0.001) and TNF-α (*p* < 0.001) in mice with PD (Figure 3F–I). Similarly, exercise was again found to effectively alleviate the inflammatory state in these mice (Iba-1, *p* = 0.043; IL-1β, *p* = 0.007; TNF-α, *p* = 0.004). Taken together, the results suggest that exercise has a significant beneficial effect on microglial activation and the expression of inflammatory factors.

### 3.4. Exercise Modulates Microglial Transition Toward Anti-Inflammatory Functional States

**Figure 4:**
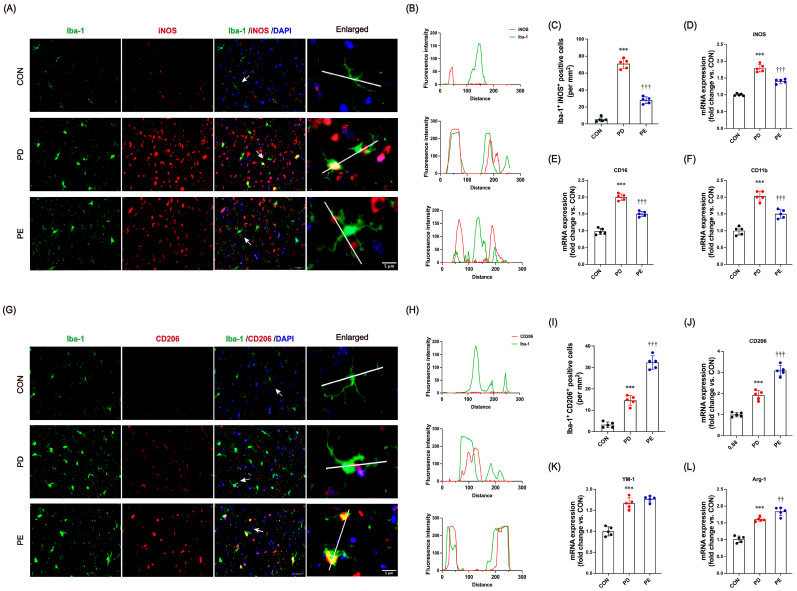
(**A**) Images of co-staining of Iba-1 (green), iNOS (red), and DAPI (blue) (scale bars, 100 μm), and enlargements (scale bars, 50 μm). The white line in the enlarged microphotographs denotes a 260-pixel cross-section for quantitative analysis of fluorescence intensity (green for microglia, red for inflammatory factors), the arrow indicates the region of interest for the zoomed-in view. (**B**) Fluorescence density analysis of Iba1^+^iNOS^+^ co-localized cells images. (**C**) The percentage of co-localized areas of Iba1^+^iNOS^+^ cells. (**D**) Images of co-staining of Iba-1 (green), CD206 (red), and DAPI (blue) (scale bars, 100 μm; enlarged images, 50 μm). (**E**) Fluorescence density analysis of Iba1^+^CD206^+^ co-localized cell images. (**F**) The percentage of co-localized areas of Iba1^+^CD206^+^ cells. (**G**–**I**) mRNA levels, including iNOS, CD16, and CD11b. (**J**–**L**) mRNA levels, including CD206, YM-1, and Arg-1. All immunofluorescence images were processed with uniform brightness/contrast adjustments using ImageJ software without altering the original data interpretation. *** represent *p* < 0.001 when comparing with the CON group; ^††^ and ^†††^ indicate *p* < 0.01 and *p* < 0.001, respectively, in comparison with the PD group. CON, control; PD, Parkinson’s disease; PE, PD plus exercise.

Recent research has shown that PD tends to promote an activated state of microglia that aims to eliminate threats and restore homeostasis [15,16]. The ability of exercise to have a positive impact on PD and its symptoms is thought to be related to its anti-inflammatory effects. We therefore hypothesized that exercise might modulate microglial functional states toward a protective phenotype and influence their interactions with neurons and other glia. To test this, we analyzed the proportion of microglia expressing pro-inflammatory (iNOS) and anti-inflammatory (CD206) markers in the SN by co-localization with Iba-1. Compared to the controls, PD mice showed increased expression of both iNOS and CD206, indicating a mixed microglial activation profile. Co-localization analysis revealed a strong overlap of Iba-1 with both iNOS (Figure 4A–C, *p* < 0.001) and CD206 (Figure 4G–I, *p* < 0.001) in MPTP-injected mice. Notably, exercise significantly reduced Iba-1^+^iNOS^+^ cells (*p* < 0.001) while increasing Iba-1^+^CD206^+^ cells (*p* < 0.001), suggesting that exercise shifts microglial states toward anti-inflammatory programs in mice with PD.

Additionally, RNA samples from the SN were analyzed to assess gene expression associated with microglial functional states. Compared to the controls, PD mice exhibited increased expression of pro-inflammatory markers iNOS (*p* < 0.001), CD16 (*p* < 0.001), and CD11b (*p* < 0.001) (Figure 4D–F) and decreased anti-inflammatory markers CD206 (*p* < 0.001), YM-1 (*p* < 0.001), and Arg-1 (*p* < 0.001) (Figure 4J–L, *p* < 0.001), indicating co-expression of both inflammatory and reparative programs. Following 10 weeks of treadmill exercise, expression of iNOS (*p* < 0.001), CD16 (*p* < 0.001), and CD11b (*p* < 0.001) decreased significantly (*p* < 0.001), while CD206 (*p* < 0.001) and Arg-1 (*p* = 0.007) levels increased, suggesting that exercise shifts microglial states toward anti-inflammatory profiles. These findings support a model where exercise modulates microglial plasticity along a dynamic continuum in PD mice.

### 3.5. Exercise Promotes AMPK/Sirt1 Signaling, but This Is Blocked by the Irisin Receptor Inhibitor

**Figure 5:**
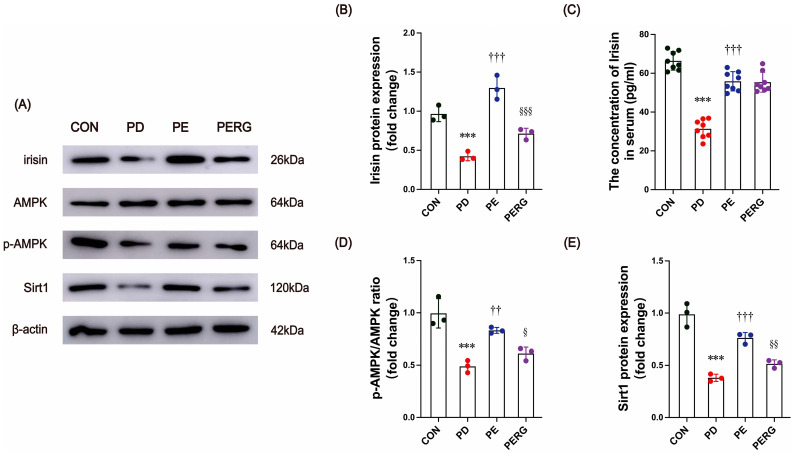
(**A**) Western blot image of irisin, AMPK, p-AMPK, and Sirt1 protein in mice substantia nigra. (**B**) Quantitative analysis of irisin protein. (**C**) Serum levels of irisin. (**D**) Quantitative analysis of p-AMPK/AMPK ratio. (**E**) Quantitative analysis of Sirt1 protein. *** represent *p* < 0.001 when comparing with the CON group; ^††^ and ^†††^ indicate *p* < 0.01 and *p* < 0.001, respectively, in comparison with the PD group; ^§^, ^§§^, and ^§§§^ denote *p* < 0.05, *p* < 0.01, and *p* < 0.001, respectively, compared with the EX group. CON, control; PD, Parkinson’s disease; PE, PD plus exercise; PERG, PD plus exercise and cycloRGDyk.

Previous research has shown that engaging in exercise can have anti-inflammatory effects and improve brain function. This is achieved mainly through the activation of various molecules that have positive impacts on neuropathology, such as the reduction in Aβ amyloid and Tau protein deposition [51]. Among these factors, irisin, AMPK, and Sirt1 are most closely associated with exercise training and affect brain plasticity and function. We further examined these molecule expressions in PD mice, and the results showed that irisin expression exhibited a marked decrease in both the serum and SN following the injection of MPTP (Figure 5A–C, *p* < 0.001). The expression of AMPK and its downstream protein Sirt1 decreased in the SN (Figure 5A,D,E, *p* < 0.001). Exercise significantly increased serum levels of irisin and its protein expression in the SN of PD mice (Figure 5A–C, *p* < 0.001). Similarly, the expression of AMPK and the Sirt1 protein was elevated by 10-week treadmill running. Early studies indicated the exercise-induced enhancement of AMPK (*p* = 0.004) and Sirt1 (*p* < 0.001) by increasing irisin signaling pathways [23]. We determined whether the blocking of the irisin receptor pathway affected brain levels of AMPK and Sirt1. Ten weeks of injections of cycloRGDyk, a putative irisin receptor inhibitor, significantly reduced the brain levels of AMPK (*p* = 0.047) and Sirt1 (*p* = 0.009), indicating that their production was under the modification of irisin signaling.

### 3.6. Blocking Irisin Pathways Could Diminish the Exercise-Induced Neuroprotective Effects on PD

**Figure 6:**
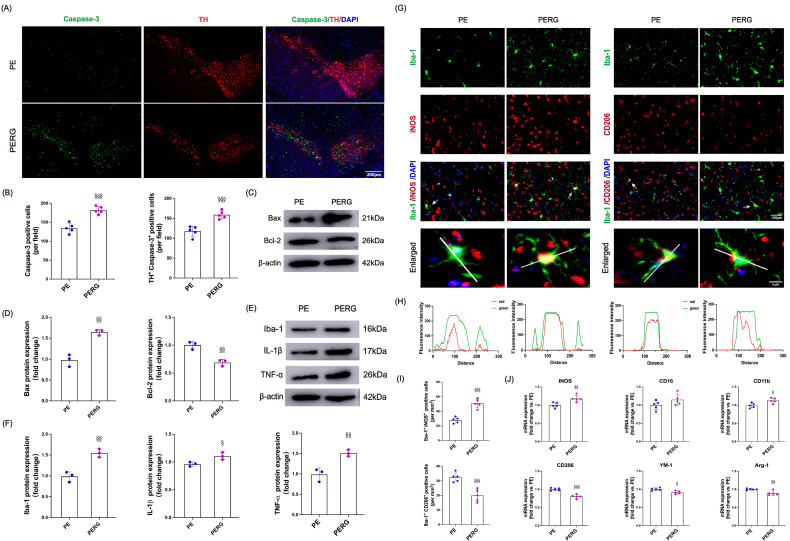
(**A**) Representative images and enlargements of fluorescent double staining of TH (red) and caspase-3 (green) (scale bars, 200 μm). (**B**) Quantitative analysis of caspase-3 staining and number of co-localized TH^+^ caspase-3^+^ cells in the substantia nigra (per field). (**C**,**D**) Western blot bands of Bax and Bcl-2 protein and quantitative analysis. (**E**,**F**) Western blot bands of Iba-1, IL-1β, and TNF-α protein and quantitative analysis. (**G**–**I**) Co-staining of Iba-1 (green), iNOS/CD206 (red), and DAPI (blue) (scale bars, 100 μm), and enlarged images (scale bars, 50 μm), as well as relative quantitative analysis. The white line in the enlarged microphotographs denotes a 260-pixel cross-section for quantitative analysis of fluorescence intensity (green for microglia, red for inflammatory factors), the arrow indicates the region of interest for the zoomed-in view. (**J**) mRNA levels, including iNOS, CD16, CD11b, CD206, YM-1, and Arg-1. All immunofluorescence images were processed with uniform brightness/contrast adjustments using ImageJ software without altering the original data interpretation. ^§^, ^§§^, and ^§§§^ denote *p* < 0.05, *p* < 0.01, and *p* < 0.001, respectively, compared with the EX group. CON, control; PD, Parkinson’s disease; PE, PD plus exercise; PERG, PD plus exercise and cycloRGDyk.

As mentioned above, the inhibition of irisin signaling led to a decrease in AMPK and Sirt1 expression in the brain, which is involved in the effects of exercise. We further tested whether inhibiting irisin pathways could affect the beneficial effects of exercise on neuroinflammation, microglia activation, and apoptosis in PD mice. For apoptosis, when compared to the PE group, mice in the PERG group showed a significant increase in caspase-3-positive cells and TH^+^ caspase-3^+^ co-localized cells (Figure 2A,B, *p* < 0.001), accompanied by upregulated Bax (*p* = 0.002) and downregulated Bcl-2 (*p* = 0.004) protein expression (Figure 6C,D). For neuroinflammation, quantitative analysis confirmed the significant upregulation of Iba-1 (*p* < 0.004), IL-1β (*p* = 0.031), and TNF-α (*p* = 0.007) protein levels in the PERG group relative to the PE group (Figure 6E,F). Regarding microglial activation, 10 weeks of cycloRGDyk intervention led to a significant increase in the intensity and number of Iba-1^+^iNOS^+^ co-localized cells and a concomitant decrease in Iba-1^+^CD206^+^ cells (Figure 6G–I, *p* < 0.001). Additionally, mice in the PERG group exhibited elevated mRNA levels of the pro-inflammatory markers iNOS (*p* = 0.009) and CD11b (*p* = 0.017) and reduced levels of the anti-inflammatory markers CD206 (*p* < 0.001), YM-1 (*p* = 0.011), and Arg-1 (*p* < 0.007) (Figure 6J). Collectively, these findings highlight the essential role of irisin signaling in mediating exercise-induced neuroprotection against apoptotic, inflammatory, and microglial dysregulation in PD mice.

## 4. Discussion

Our study shows that physical exercise can produce multiple positive effects on neuropathology in PD mice. These benefits include the following: (1) Slowing down the gradual loss of dopaminergic neurons, which is associated with improved locomotor function. (2) Decreasing excessive apoptosis and neuroinflammation, and promoting microglial transition toward anti-inflammatory functional states. (3) Irisin is a crucial factor in the neuroprotection related to exercise intervention, and the AMPK/Sirt1 signaling pathway is involved in this process.

PD is often considered incurable due to its irreversible pathological process. As a result, treatment options mainly focus on symptom management and improving the patient’s quality of life [1]. Exercise is a favorable treatment option that can help individuals with PD cope with their condition and maintain their physical and mental health [52,53]. Recent evidence from both human [54,55,56] and animal [19,57,58] studies has shown that exercise can effectively alleviate neurodegenerative processes and improve PD symptoms. Our findings are consistent with previous studies, demonstrating that exercise improves motor balance function and significantly reduces the loss of dopaminergic neurons in PD mice. Thus, exercise can be considered a good medicine for PD.

Exercise can bring multiple benefits to PD pathology, one of which is the alleviation of neuroinflammation. Dysregulated inflammation is a significant contributor to persistent neurodegeneration, nerve cell death, and the worsening of neurological consequences in PD [59].

The activation states of microglia, historically categorized as M1 and M2 phenotypes, play a crucial role in regulating neuroinflammation and neuronal apoptosis in response to brain injury or abnormal protein accumulation [60]. However, emerging evidence emphasizes that microglia exist along a dynamic functional continuum rather than as fixed phenotypes [11]. Studies suggest that modulating microglial transitions toward anti-inflammatory programs can effectively improve neural network homeostasis and ameliorate PD pathology [15,16,42,61,62,63,64]. This approach holds promise for alleviating neuroinflammation in neurological disorders. Our findings demonstrate that exercise reduces pro-inflammatory microglial states while promoting anti-inflammatory microglial programs in PD mice. We also observed significant improvements in the abnormal expression of inflammation-associated factors and apoptosis-associated markers. These findings support the protective and therapeutic effects of exercise against neuroinflammation and neuronal apoptosis in PD.

Recent evidence suggests that irisin is a pivotal myokine that mediates interactions between exercise and multiple organs, such as muscle–bone crosstalk and muscle–brain crosstalk [23,25]. The identification of irisin in different cerebral areas and its capability of mediating favorable impacts on the brain after exercise intervention [65] indicates that irisin may be a crucial molecule involved in exercise-induced neuroprotective effects. Studies have revealed a notable decrease in FNDC5/irisin expression in PD [66,67], and irisin treatment has been found to prevent apoptosis and degeneration of DA neurons [66,68]. Our study also found that circulation levels and brain expression of irisin in PD mice were reduced. More interestingly, a 10-week exercise protocol could effectively reverse the reduction in serum and brain levels of irisin. This upregulation of irisin levels may be involved in the exercise-related neuroprotective effects in PD mice because after blocking irisin receptor signaling (αV/β5), the exercise-induced benefits were inhibited. αV/β5 is generally recognized as a receptor for irisin, and its inhibitor, cycloRGDyk, can effectively block irisin signaling [37]. Additionally, cycloRGDyk (molecular weight: 847.7 g/mol) could cross the BBB and, therefore, might inhibit both peripheral and central irisin signaling. According to a recent study by Zhang et al. [69], irisin was found to bind to the integrin V5 receptor on microglia, which constitutes a significant portion of the total V integrins expressed by these cells. Wrann’s study showed that exercise led to an increase in FDNC5/irisin expression in the rodent brain, which promoted the production of BDNF [70]. Irisin also has anti-inflammatory effects, such as reducing the NOD-like receptor protein 3 (NLPR3) inflammasome and reactive oxygen species production [71]. Moreover, administering irisin to astrocytes helped protect neurons from Aβ toxicity and led to a decrease in the production of interleukin-6 (IL-6) and IL-1β as well as a reduction in the nuclear factor kappa-light-chain-enhancer of activated B cells (NF-kB) [72]. Together, these reports, as well as our findings, suggest that irisin may be a critical molecule mediating the beneficial effects of exercise on PD by alleviating its neuroinflammatory status.

A growing body of research indicates that irisin and other molecules, such as AMPK and Sirt1, work together to regulate metabolic and immune responses to physical exercise [73,74]. AMPK activation has been found to have a significant impact on the neuroinflammatory function of microglia/macrophage cells and modulate the polarization phenotype of immune cells, acting as a potent protective regulator [74]. Wang et al. [31] demonstrated that irisin mitigates neuroinflammation and apoptosis in intracerebral hemorrhage mice by activating αV/β5/AMPK signaling, which orchestrates the microglial transition toward reparative functional states. Sirt1 has beneficial roles in neuroinflammation-related diseases, such as ischemic stroke, traumatic brain injury, spinal cord injury, AD, and PD [75]. It can modulate various cellular processes, such as apoptosis, DNA repair, inflammatory response, metabolism, and stress [75]. AMPK and Sirt1 are interconnected in a positive feedback loop [74]. Therefore, irisin together with AMPK and Sirt1 may contribute to inhibiting neuroinflammation. Our results are in accordance with this notion, revealing that exercise rescued the decreased levels of AMPK phosphorylation and Sirt1 expression in PD mice, whereas these beneficial changes were inhibited by blocking irisin signaling. Moreover, blocking irisin pathways also led to increased inflammation and mass apoptosis in PD mice. Together, this implied that the irisin/AMPK/Sirt1 signaling axis is involved in the neuroprotective effects of exercise against PD.

## 5. Conclusions

This study demonstrates that 10 weeks of treadmill exercise exerts neuroprotective effects in MPTP-induced PD mice by reducing microglia-mediated neuroinflammation, alleviating dopaminergic neurodegeneration, and improving motor function. Mechanistically, exercise promotes the microglia’s conversion from a pro-inflammatory to anti-inflammatory phenotype, which is associated with upregulated irisin expression and activation of the AMPK/Sirt1 signaling pathway. Blocking the irisin receptor with cycloRGDyk abolishes these beneficial effects, confirming the central role of the irisin/AMPK/Sirt1 axis in exercise-induced neuroprotection.

This study has several limitations. First, it included only male mice, restricting insights into sex differences in PD pathology. Future studies will incorporate both male and female mice. Second, all experiments were conducted exclusively on young male mice (8 weeks old). While young animals are commonly used in PD research [13,15,16,76], including older mice could provide more comprehensive data. Additionally, this study conducted only in vivo experiments. In contrast, ex vivo cell cultures may offer a deeper understanding of regulatory mechanisms. Future investigations should involve larger cohorts (including females and older populations), utilize advanced techniques such as high-resolution morphological analyses, and employ mechanistic models like microglial depletion, cell cultures, and tracers to better understand the effects of exercise on PD and its underlying mechanisms.

## Figures and Tables

**Table 1:** The sequences of primers used for RT q-PCR.

Gene	Primer	Sequence
*iNOS*	Forward	5′-CTGCCCCCCTGCTCACTC-3′
	Reverse	5′-TGGGAGGGGTCGTAATGTCC-3′
*CD16*	Forward	5′-TTTGGACACCCAGATGTTTCAG-3′
	Reverse	5′-GTCTTCCTTTGAGCACCTGGATC-3′
*CD11b*	Forward	5′-GAGCAGCACTGAGATCCTGTTTAA-3′
	Reverse	5′-ATACGACTCCTGCCCTGGAA-3′
*Arg1*	Forward	5′-GAACACGGCAGTGGCTTTAAC-3′
	Reverse	5′-TGCTTAGCTCTGTCTGCTTTGC-3′
*YM-1*	Forward	5′-AGGAAGCCCTCCTAAGGACAAACA-3′
	Reverse	5′-ATGCCCATATGCTGGAAATCCCAC-3′
*CD206*	Forward	5′-AAGGAAGGTTGGCATTTGT-3′
	Reverse	5′-CCTTTCAATCCTATGCAAGC-3′
*GAPDH*	Forward	5′-TTCAACGGCACAGTCAAGGC-3′
	Reverse	5′-GACTCCACGACATACTCAGCACC-3′

## Data Availability

The corresponding author will provide the necessary data supporting the findings of this study upon a reasonable request. The authors are accountable for ensuring the continued availability of the data.

## Supplementary Materials

The following supporting information can be downloaded at: https://www.mdpi.com/article/10.3390/biology14080955/s1, Figure S1: Negative control images of TH immunohistochemical staining in the substantia nigra and striatum regions.
