# Supplementary material for: Exercise Ameliorates Dopaminergic Neurodegeneration in Parkinson’s Disease Mice by Suppressing Microglia-Regulated Neuroinflammation Through Irisin/AMPK/Sirt1 Pathway
Source: Biology (Basel). 2025 Jul 29;14(8):955. doi: 10.3390/biology14080955 (PMC12383941; doi:10.3390/biology14080955)
Supplement: Supplementary file 1 [file biology-14-00955-s001.zip › biology-3618369-supplementary.pdf]

(A)

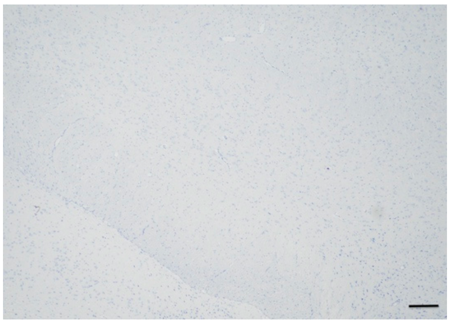

(B)

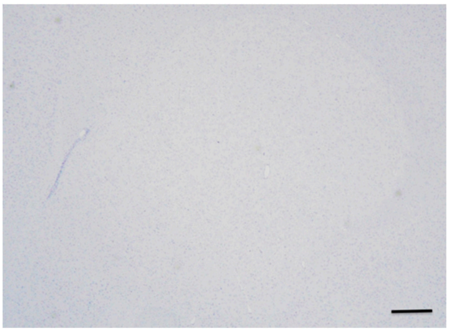

(A) Immunohistochemical negative control for substantia nigra region, scale bar, 200 μm;
(B) Immunohistochemical negative control for striaum, scale bar, 500 μm.
